# Factors associated with patient empowerment in Spanish adults with type 2 diabetes: A cross‐sectional analysis

**DOI:** 10.1111/hex.13501

**Published:** 2022-09-01

**Authors:** Andrea Duarte‐Díaz, Himar González‐Pacheco, Amado Rivero‐Santana, Yolanda Ramallo‐Fariña, Lilisbeth Perestelo‐Pérez, Wenceslao Peñate, Carme Carrion, Pedro Serrano‐Aguilar, INDICA Team

**Affiliations:** ^1^ Canary Islands Health Research Institute Foundation (FIISC) Tenerife Spain; ^2^ Department of Clinical Psychology, Psychobiology and Methodology University of La Laguna (ULL) Tenerife Spain; ^3^ Research Network on Health Services in Chronic Diseases (REDISSEC) Tenerife Spain; ^4^ Network for Research on Chronicity, Primary Care, and Health Promotion (RICAPPS) Tenerife Spain; ^5^ Evaluation Unit (SESCS) Canary Islands Health Service (SCS) Tenerife Spain; ^6^ eHealth Lab Research Group, School of Health Sciences Universitat Oberta de Catalunya (UOC) Barcelona Spain

**Keywords:** correlates, patient empowerment, Spain, type 2 diabetes mellitus

## Abstract

**Objective:**

The aim of the present study is to identify factors associated with patient empowerment in people living with type 2 diabetes mellitus (T2DM) in the Canary Islands (Spain).

**Methods:**

Secondary cross‐sectional analysis was carried out of data obtained in the INDICA study: A 24‐month cluster randomized‐controlled trial evaluating the effectiveness of educational interventions supported by new technology decision tools for T2DM patients. Sociodemographic variables, clinical data (years since diagnosis, glycated haemoglobin level, creatine, triglycerides, waist hip index, body mass index and number of comorbidities), diabetes knowledge (DIATEK), affective outcomes (Beck Depression Inventory‐II, the State subscale of the State‐Trait Anxiety Inventory and The Diabetes Distress Scale) and diabetes‐related quality of life (The Audit of Diabetes‐Dependent Quality of life) were assessed as potential correlates of patient empowerment, assessed using the Diabetes Empowerment Scale‐Short Form. Multilevel mixed linear regression models on patient empowerment were developed.

**Results:**

The analysis included the baseline data of 2334 patients. Results showed that age (*B* = −0.14; *p* < .001), diabetes knowledge (*B* = 0.61; *p* < .001) and state‐anxiety (*B* = −0.09; *p* < .001) are significantly associated with patient empowerment. Sex, education level, living alone, employment status, country of birth, time since diagnosis, number of comorbidities, glycated haemoglobin level, depression and distress were not independently associated with patient empowerment in the multivariate analyses.

**Conclusion:**

Younger age, lower state‐anxiety and greater diabetes‐specific knowledge are important correlates of patient empowerment. In line with the results of the INDICA study, interventions based on patient‐centred care might be effective in improving patient empowerment in adults with T2DM. Understanding the factors associated with empowerment may help clinicians and policymakers to identify high‐risk groups, prioritize resources and target evidence‐based interventions to better support people with T2DM to be actively involved in their own care.

**Patient or Public Contribution:**

Patients with T2DM were actively involved in the design of the INDICA study. Two patient associations were included as part of the research team and actively participated in designing the interventions and selecting outcome measures.

## 1 INTRODUCTION

Diabetes Mellitus (DM) is one of the most common diseases in the modern world, and its burden^1^ and prevalence have steadily increased over the last decade.^2^, ^3^ In 2017, the International Diabetes Federation estimated that 451 million people aged 18–99 years were suffering from diabetes worldwide.^4^ More recently, these figures have been updated and the global prevalence of diabetes in 2019 was estimated to be 9.3% (463 million people), with an expected rise to 10.2% (578 million) by 2030 and 10.9% (700 million) by 2045 if no effective prevention strategies are adopted.^5^ Type 2 diabetes mellitus (T2DM), which accounts for 90%–95% of all cases of diabetes, has a substantial impact on health‐related quality of life and socioeconomics.^6^, ^7^ Its specific incidence and prevalence have also increased around the world, with higher rates in low‐middle, middle and high‐middle socioeconomic development countries.^1^ In Spain, the overall incidence of T2DM has been estimated to be 11.6 cases/1000 person‐year.^8^ National studies have confirmed the significant negative impact that T2DM has on both health‐related quality of life and affective outcomes. For instance, a cross‐sectional multicentre study comparing T2DM patients with the general population found that health‐related quality of life was worse among diabetic patients.^9^ Similarly, a large prospective cohort study found that depression was highly prevalent in Spanish T2DM patients and subsequently associated with several key diabetes‐related outcomes.^10^ In the case of the Canary Islands, complications of T2DM and its related mortality are higher than those in other Spanish regions.^11^, ^12^ A descriptive study of 40.392 major amputations among patients with T2DM in different Spanish regions recently showed a growing trend in the Canary Islands, which diverged from the global downward trend in Spain.^12^

As T2DM is a chronic condition, patients are required to incorporate multiple lifestyle changes in their everyday life. Frequently, this implies embracing new routines and habits in relation to diet, physical activity and treatment adherence. Self‐care is an essential component of diabetes care. However, a significant proportion of patients fail to engage in adequate self‐management and show major difficulties in maintaining treatment adherence.^13^, ^14^ Therefore, promoting diabetes self‐management has been widely recommended.^15^

Person‐centred care (PCC) implies the consideration of the patient as a whole person, respecting their autonomy and incorporating their values, preferences, affective states and specific circumstances into the decision‐making process about their health.^16^ To optimize this process, professionals must not only provide good‐quality clinical information but also promote patients' capacity and motivation to be actively involved in the decisions about their treatment and in the self‐management of their disease.^17^, ^18^ Contrary to a patient considered as a mere recipient of experts' therapeutic procedures who must comply with their indications, PCC aims to increase the patients' empowerment to actively and successfully manage their disease. Although a consensus definition of patient empowerment is still lacking and its meaning overlaps with other constructs like self‐efficacy or patient activation,^19^, ^20^ it can be considered as a process where patients are motivated and supported to be responsible for their care and to take the management of their condition into their own hands.^16^ Accordingly, an empowered patient is one who has the knowledge, motivation and capacity to take control of his or her own care. Current evidence suggests that activated patients are more likely to adhere to treatment plans and lifestyle modification.^21^, ^22^ However, patient empowerment not only refers to those psychological aspects related to motivation, behavioural change and adherence to self‐care strategies but also involves a process of psychological transformation and acceptance in which the reality of illness becomes part of one's identity.^23^, ^24^ Accordingly, patient empowerment can be conceptualized as a continuous developmental and psychological transition from an identity threatened by the presence of the disease, to an empowered one that integrates disease reality.^23^

While the positive effect of empowerment on clinical and psychosocial outcomes seems to be established,^25^, ^26^, ^27^, ^28^ less is known about its correlates. To better understand patients' willingness to be actively involved in their care, it is essential to explore which factors could lead them to feel empowered. Identification of personal and clinical factors related to patient empowerment might enable health‐care professionals to recognize those patients with T2DM who could benefit more from an empowerment‐based intervention, or even adjust the complexity of the available interventions to the estimated probabilities of patients' success. Likewise, determining those psychosocial factors that play an important role in patient empowerment could make the difference when designing interventions to promote patient involvement.

The *Diabetes Intervention Study In The Canary Islands* (INDICA) was a 24‐month multiarm cluster randomized‐controlled trial aimed at assessing the effectiveness and cost‐effectiveness of three multicomponent interventions based on the conceptual framework of behavioural change and PCC.^29^, ^30^, ^31^ The interventions combined educational groups and training on patient empowerment and PCC with different information and communication technology‐based interventions to guide the decisions of the main actors involved in the management of T2DM.^29^, ^30^, ^31^

As engaging stakeholders in the codevelopment of the interventions was a priority in the INDICA study, both healthcare professionals and patients played an active role. Primary care professionals participated in the elaboration of the study protocol, and two groups of patients with T2DM were included as part of the research team and actively participated in the design of the interventions and selection of the outcomes. The primary endpoint was the mean change in glycated haemoglobin from baseline to the 24‐month follow‐up. Among the secondary measures, different cognitive‐attitudinal, behavioural and affective outcomes were also assessed, including patient empowerment. The aim of the present study is to identify factors associated with patient empowerment in people living with T2DM in the Canary Islands (Spain).

## 2 METHODS

### 2.1 Study design

This cross‐sectional analysis used the baseline data from the INDICA study (ClinicalTrials identifier NCT01657227). More details about the INDICA study design have been reported in a previous publication,^29^ and the results on effectiveness can be reviewed in Ramallo‐Fariña et al.^30^, ^31^ This manuscript was written in accordance with the STROBE statement for cross‐sectional studies^32^ (File S1). The scientific and ethics committee of the University Hospital Nuestra Señora de la Candelaria approved the study protocol (ID: EPA‐07/10). The study was performed in accordance with Good Clinical Practice standards, applicable local regulatory requirements and the Code of Ethics of the Declaration of Helsinki.

### 2.2 Setting and recruitment

The INDICA study was conducted in four of the eight Canary Islands (Tenerife, Gran Canaria, Lanzarote and La Palma). First, primary healthcare practices (PHCPs) were recruited. Only PHCPs with at least eight Family Care Units (FCUs) and availability of appropriate places to provide group sessions were included. Local health authorities and PHCP directors were contacted and invited to an informative meeting, where the main study objectives, time frames and tasks were described. A list of all the available FCUs, composed of a family physician and a nurse, in every selected PHCP was provided and they were invited to participate. FCUs were randomly selected from all those consenting to participate in each PHCP. After that, potential eligible patients in each selected FCU were identified through the electronic clinical records. Once identified, the study staff contacted patients by phone and invited them to a face‐to‐face meeting, where they could receive detailed information about the study. Finally, patients were selected from all those who fulfilled the inclusion criteria and provided informed consent.

### 2.3 Participants

Participants were recruited in 2015 from 32 PHCPs. Eligible participants were patients with T2DM diagnosed at least 1 year before study enrolment, without severe comorbidities and whose age ranged from 18 to 65 years. The complete inclusion and exclusion criteria for patients and PHCP are shown in Table 1. All participants of the INDICA study provided written informed consent.

**Table 1:** Inclusion and exclusion criteria

Inclusion criteria	Patients
	Patients with T2DM diagnosed at least 1 year before study enrolment. 18–65 years of age. Formal consent to participate in the study. Regular use of mobile phone. PHCP: At least eight Family Care Units. Availability of appropriate places to provide group sessions.
Exclusion criteria	Patients
	Chronic kidney disease ≥ Stage 3, as defined by the National Kidney Foundation's Kidney Disease Outcomes and Quality Improvement Initiative urinary albumin to creatinine ratio ≥300 mg/g, and/or urinary protein excretion ≥300 mg/24 h. Acute coronary syndrome (documented angina or myocardial infarction) or stroke in the last 6 months or Class III or IV heart failure, according to the New York Heart Association. Proliferative diabetic retinopathy or clinically significant diabetic macular oedema requiring previous treatment with retinal photocoagulation, vitrectomy or intravitreal injections of antivascular endothelial growth factor or triamcinolone acetonide 6 months before study inclusion. Uncorrected severe hearing or visual impairment or corrected visual acuity ≤20/40 by any cause. Diabetic foot with ulcers ≥2 according to the Wagner scale. Liver cirrhosis. Cancer unless disease cancer‐free 5 years after diagnosis. Other terminal illnesses. Intellectual disability, dementia or psychotic diseases. Active substance abuse. Pregnancy. Insufficient Spanish language skills. Physical disability limiting participation in group education activities. Concurrent participation in another clinical trial or any other investigational study. PHCP Less than eight Family Care Units. Places to provide group sessions not available.

Abbreviations: PHCP, primary healthcare practices; T2DM, type 2 diabetes mellitus.

### 2.4 Data collection and measures

#### 2.4.1 Outcome variable: Patient empowerment

Patient empowerment was assessed using the Diabetes Empowerment Scale‐Short Form (DES‐SF),^33^ an eight‐item short form of the original DES, developed by Anderson et al.,^34^ to measure perceived diabetes‐related self‐efficacy. Each of the eight items of the DES‐SF is rated on a 5‐point Likert scale ranging from ‘strongly disagree’ to ‘strongly agree’. Total score ranges from 8 to 40, and higher scores indicate a stronger level of patient empowerment. DES‐SF has shown good internal consistency (Cronbach's *α* coefficient of .83) and stability over time (*r* = .532; *p* = .009).^35^ The DES‐SF has been translated and adapted for Spanish‐speaking older adults with chronic diseases, replacing references to ‘diabetes’ with the word ‘health’ so that it can be applied to all kinds of health conditions.^36^ This adaptation has shown good internal consistency (Cronbach's *α* of .89) and convergent validity with the General Self Efficacy Scale (*r* = .77).^36^

#### 2.4.2 Potential correlates

2.4.2.1

*Sociodemographic data*. Sociodemographic data included age, gender, educational level (primary or lower education vs. secondary or higher education), living alone (yes vs. no), marital status (with a partner vs. without a partner), employment status (active vs. nonactive), country of birth (Spain vs. out of Spain) and monthly income (<500€ vs. ≥500€).

2.4.2.2

*Clinical data*. Clinical data included years since T2DM diagnosis, glycated haemoglobin (HbA1c) level (<8% and ≥8%), creatine, triglycerides, waist hip index, body mass index (normal‐underweight [<25], overweight [<30], Class I obesity [<35], class II obesity [<40] and class III obesity [≥40]) and number of comorbidities. In the INDICA study, randomization was performed, first creating strata according to geographical areas and then randomly allocating PHCPs (clusters) to every geographical stratum. Thus, in this cross‐sectional analysis, because of the cluster nature of the data, the intraclass correlation (ICC) was controlled by entering PHCPs in the models as random effects.

2.4.2.3

*Diabetes knowledge*. The Diabetes Knowledge Test (DIATEK) (Material S2), a specific instrument created in the context of the INDICA project, was used to assess knowledge about T2DM. It consists of 30 items with four response options and only one correct answer. Items examined risk factors for disease development and deterioration, objective values for biochemical parameters and recommendations on nutrition, physical activity, medication and self‐management. The total score, ranging from 0 to 30, was obtained through summation of all correct responses. This score was later rescaled from 0 to 10.

*Affective variables*:

1. Depression level was assessed using the Beck Depression Inventory‐II,^37^ a 21‐item self‐reported validated measure to evaluate depressive symptoms and quantify their severity. Each item is rated in a 4‐point Likert scale (from 0 to 3), resulting in a total score ranging from 0 to 63. According to the authors, a score of 0–13 indicates minimal or no depression; a score of 14–19 indicates mild depression; a score of 20–28 indicates moderate depression and a score of 29–63 indicates severe depression. Studies on its psychometrical properties have reported an average Cronbach's *α* of around .9 and good to excellent retest reliability coefficients (range: 0.73–0.96).^38^ This inventory has been adapted to different languages, including Spanish. The Spanish adaptation has shown good internal consistency in general,^39^ medical^40^ and psychiatric samples^41^ (Cronbach's *α* of .87, .92 and .89, respectively).
2. The state subscale of the State‐Trait Anxiety Inventory (STAI)^42^ was used to assess a transient emotional state characterized by perceived subjective feelings of tension and apprehension. The STAI is a psychological self‐reported inventory and consists of 40 questions, 20 for trait‐anxiety (STAI‐T) and 20 for state‐anxiety (STAI‐S). Each item must be rated on a 4‐point Likert scale from 0 (*not at* all) to 3 (*very much so*). Items are summed to obtain a total score ranging from 0 to 60, with higher scores reflecting greater anxiety. This inventory has been adapted in Spanish,^43^ and a psychometric revision found Cronbach's *α* of .90 and .94 for trait and state subscales, respectively.^44^
3. The Diabetes Distress Scale (DDS2),^45^ a validated distress screening tool, was used to assess the degree of perceived diabetes‐related distress. It is available in English and Spanish versions, and comprises two items (*feeling overwhelmed by the demands of living with diabetes* and *feeling that I am often failing with my diabetes regimen*) ranging on a six‐point Likert scale from 1 (*not a problem*) to 6 (*serious problem*). The total score is based on the sum of individual scores from each item, and accordingly, three categories can be defined: little or no distress (<2), moderate distress (2.0–2.9) and high distress (≥3).^46^ Properties for the DDS2 are similar to the longer DDS17 version, and good internal consistency has been found (Cronbach's *α* of .84).^45^, ^47^

2.4.2.4

*Diabetes‐related quality of life*. The Audit of Diabetes‐Dependent Quality of life (ADDQoL)^48^ was used to measure health‐related quality of life. ADDQoL assess 19 domains related to the impact of diabetes on specific aspects of life: leisure activities, working life, local or long‐distance journeys, holidays, physical health, family life, friendships and social life, close personal relationships, sex life, physical appearance, self‐confidence, motivation to achieve things, people's reactions, feelings about the future, financial situation, living conditions, dependence on others, freedom to eat and freedom to drink. First, for each domain, both impact and attributed importance are rated. The scales range from −3 to +1 for impact rating and from 0 to 3 for importance rating. Then, a weighted score for each domain is calculated as a multiplier of impact rating and importance rating. This weighted total score ranges from −9 to +3, and lower scores indicate poorer quality of life. The original ADDQoL has shown good internal consistency, with Cronbach's *α* above .90,^48^, ^49^, ^50^ and also the Spanish adaptation.^51^

#### 2.4.3 Sample size

Sample size was calculated for the primary outcome of the cluster randomized‐controlled trial. It was estimated that 1,572 patients were needed to detect an absolute difference in HbA1C of 0.4%, assuming a common standard deviation of 1.4%,^52^ a two‐tailed power of 90% and an *α* of .05. After adjusting for clustering of patients and an additional 30% increase to accommodate for expected losses to follow‐up, a total sample of 2330 was calculated. Although the sample size was not fixed for this secondary analysis, our sample size was high enough according to the rule of thumb of a minimum of 10 participants per predictor variable.

#### 2.4.4 Statistical methods

Statistical analyses were performed using STATA 15.0 software. The main characteristics of the sample and the study variables were described using frequencies and percentages for categorical variables and mean and standard deviation for continuous variables. The association between patient empowerment and its potential correlates was assessed in a bivariate analysis. The bivariate associations with demonstrated levels of statistical significance (*p* < .05) were combined into a stepwise lineal regression model. A forward strategy was used to enter variables hierarchically by blocks according to its theoretical content. The first block consisted of sociodemographic variables, the second block included clinical information, the third block included diabetes knowledge, the fourth block consisted of affective outcomes (state‐anxiety, depression and diabetes‐related distress) and the fifth block included a diabetes‐specific quality‐of‐life measure. Variables reaching a *p* < .05 level of significance remained in the model, and those with higher *p* values were removed. A multilevel mixed linear model with a random intercept was created to correct for our clustered data. In the statistical analysis, patients' characteristics were considered as first‐level variables, and PHCP was considered as a second‐level variable. Regression assumptions, such as multicollinearity and distribution, were assessed before the analysis.

## 3 RESULTS

### 3.1 Study participants

**Figure 1:**
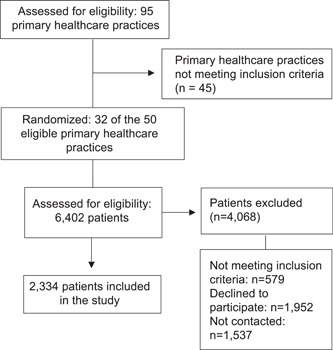
Flow diagram of the selection of participants

A total of 32 PHCPs participated in the study. A total of 6402 patients were assessed for eligibility and 2334 were finally included. Figure 1 shows the flowchart of the recruitment process. The mean age of the patients was 55.70 ± 7.14 years (range: 20–67 years), and 51.93% were females. In terms of education level, 62.47% had primary or lower education, while 37.53% had secondary or higher education. The mean time since diagnosis was found to be 8.50 ± 6.51 years, and 75.32% of patients had HbA1c levels within the accepted therapeutic goal (≤8%). Patient characteristics and empowerment scores are presented in Table 2.

**Table 2:** Participants' characteristics (*n* = 2334)

	*n* (%)	Mean (SD)
*Sociodemographic data*		
Age (years)		55.70 (7.1)
Gender		–
Male	1122 (48.1)	
Female	1212 (51.9)	
Education level		–
Primary or lower	1458 (62.5)	
Secondary or higher	876 (37.5)	
Living alone		–
Yes	209 (8)	
No	2125 (91)	
Marital status		‐
With partner	1821 (78)	
Without partner	513 (23)	
Employment status		–
Active	1367 (58.6)	
Nonactive	967 (41.4)	
Monthly income		–
<€500	1742 (74.6)	
≥€500	592 (25.4)	
Country of birth		–
Spain	2214 (94.9)	
Out of Spain	120 (5.1)	
*Clinical data*		
Years since diagnosis	–	8.50 (6.51)
HbA1c		
<8%	1758 (75.3)	7.28 (1.47)
≥8%	576 (24.7)	
BMI		
Normal‐underweight (<25)	199 (8.6)	32.07 (5.91)
Overweight (<30)	726 (31.1)	
Class I obesity (<35)	794 (34)	
Class II obesity (<40)	402 (17.2)	
Class III obesity (≥40)	213 (9.1)	
Number of comorbidities	–	2.75 (1.85)
WHI	–	0.98 (0.07)
Creatine	–	0.80 (0.18)
Triglycerides	–	162.53 (104.35)
*Patient empowerment* (DES‐SF)^33^	–	26.58 (9.17)
*Cognitive and affective outcomes*		
DIATEK	–	6.39 (1.82)
BDI‐II^37^	–	11.26 (9.90)
STAI‐S^42^	–	21.77 (13.49)
DDS2^45^	–	2.60 (1.22)
*Diabetes‐related quality of life*		
ADDQoL^48^	–	−1.80 (1.90)

Abbreviations: ADDQoL, Audit of Diabetes‐Dependent Quality of life; BDI‐II, Beck Depression Inventory‐II; BMI, body mass index; DDS2, Diabetes Distress Scale; DIATEK, Diabetes Knowledge Test; HbA1c, glycated haemoglobin; HDL, high‐density lipoprotein; LDL, low‐density lipoprotein; SD, standard deviation; STAI‐S, State subscale of the Trait Anxiety Inventory; WHI, waist hip index.

### 3.2 Correlates of patient empowerment

A multilevel stepwise linear regression analysis was conducted to identify the factors that might be significantly associated with empowerment in patients with T2DM. In terms of sociodemographic and clinical factors, age, sex, education level, living alone, employment status, country of birth, time since diagnosis, HbA1c and number of comorbidities were included in the regression models after achieving significance in the bivariate analysis. Diabetes knowledge, state‐anxiety, depression, distress and diabetes‐related quality of life were also significantly associated with patient empowerment.

In the first model, patient empowerment was significantly associated with age (*B* = −0.12; *p* < .001), while sex and education level showed a trend towards significance (*B* = −0.89; *p* = .058 and *B* = 0.90; *p* = .070, respectively). In the next step, clinical variables were included, but none reached significance. Diabetes‐related knowledge was included in the third model, and it was found to be significantly associated with patient empowerment (*B* = 0.65; *p* < .001). Next, affective outcomes (state‐anxiety, depression and diabetes‐related distress) were included. Patient empowerment was only negatively associated with state‐anxiety (*B* = −0.6; *p* = .025). The fifth model included diabetes‐related quality of life, but it did not reach statistical significance (*B* = −0.06; *p* = .636). Finally, in the full‐corrected final model, age (*B* = −0.14; *p* < .001), specific‐diabetes knowledge (*B* = 0.61; *p* < .001) and state‐anxiety (*B* = −0.09; *p* < .001) were significantly related to patient empowerment. Across all five models, ICC was low (0.03–0.036), indicating that most of the variance was attributable to within‐subject (patient level) variables. The complete multilevel stepwise linear regression analyses are shown in Table 3.

**Table 3:** Multilevel stepwise lineal regression analyses (*n* = 2334)

	Model 1: Sociodemographic			Model 2: Model 1 + clinical			Model 3: Model 2 + knowledge			Model 4: Model 3 + affective outcomes			Model 5: Model 4 + diabetes‐related quality of life			Final model		
	*B* (95% CI)	*t*	*p*‐Value	*B* (95% CI)	*t*	*p*‐Value	*B* (95% CI)	*t*	*p*‐Value	*B* (95% CI)	*t*	*p*‐Value	*B* (95% CI)	*t*	*p*‐Value	*B* (95% CI)	*t*	*p*‐Value
Age	−0.12 (−0.19; −0.06)	−3.64	**<.001**	−0.14 (−0.20; −0.07)	−4.03	**<.001**	−0.12 (−0.19; −0.06)	−3.86	**<.001**	−0.14 (−0.20; −0.07)	−4.28	**<.001**	−0.14 (−0.20; −0.08)	−4.29	**<.001**	−0.14 (−0.20; −0.07)	−4.25	**<.001**
Sex [ref: male] Female	−0.90 (−1.83; 0.02)	−1.92	.055	–	–	–	–	–	–	–	–	–	–	–	–	–	–	–
Education level [ref: primary or lower]	0.80 (−0.19;1.78)	1.59	.111	–	–	–	–	–	–	–	–	–	–	–	–	–	–	–
Secondary or higher																		
Living alone [ref: no]	−0.45 (−1.94; 1.05)	−0.58	.559	–	–	–	–	–	–	–	–	–	–	–	–	–	–	–
Yes																		
Employment status [ref: non‐active]	0.15 (−0.87; 1.18)	0.29	.772	–	–	–	–	–	–	–	–	–	–	–	–	–	–	–
Active																		
Country of birth [ref: Spain]	−1.18 (−2.95; 0.59)	−1.31	.191	–	–	–	–	–	–	–	–	–	–	–	–	–	–	–
Time since diagnosis	–	–	–	0 (−0.07; 0.07)	0	.999	–	–	–	–	–	–	–	–	–	–	–	–
Number of comorbidities	–	–	–	−0.10 (−0.34; 0.15)	−0.77	.441	–	–	–	–	–	–	–	–	–	–	–	–
HbA1c [ref: <8%] ≥8%	–	–	–	0.65 (−0.41; 1.71)	1.13	.227	–	–	–	–	–	–	–	–	–	–	–	–
DIATEK	–	–	–	–	–	–	0.65 (0.39; 0.91)	4.86	**<.001**	0.61 (0.35; 0.87)	4.57	**<.001**	0.62 (0.36; 0.89)	4.61	**<.001**	0.61 (0.35; 0.87)	4.61	**<.001**
BDI‐II^37^	–	–	–	–	–	–	–	–	–	−0.03 (−0.10; 0.04)	−0.77	.440	–	–	–	–	–	–
STAI‐S^42^	–	–	–	–	–	–	–	–	–	−0.6 (−0.12; −0.01)	−2.26	**.025**	−0.09 (−0.13; −0.05)	−4.47	**<.001**	−0.09 (−0.12; −0.05)	−4.41	**<.001**
DDS2^45^	–	–	–	–	–	–	–	–	–	0.27 (−0.78; 0.24)	−1.03	.303	–	–	–	–	–	–
ADDQoL^48^	–	–	–	–	–	–	–	–	–	–	–	–	−0.06 (−0.31; 0.19)	0.47	.636	–	–	–
Intercept	27.82 (25.75; 29.89)	26.35	**<.001**	26.41 25.47; 27.58)	62.01	**<.001**	26.57 (25.74; 27.40)	62.88	**<.001**	26.57(25.71; 27.41)	61.51	**<.001**	26.56 (25.72; 27.41)	61.77	**<.001**	26.56 (25.72; 27.41)	61.77	**<.001**
	*F* = 5.17; *p* < .001; ICC PHCP = .030			*F* = 5.67; *p* < .001; ICC PHCP = .030			*F* = 22.02; *p* < .001; ICC PHCP = .034			*F* = 11.76; *p* < .001; ICC PHCP = .036			*F* = 16.30; *p* < .001; ICC PHCP = .035			*F* = 21.22; *p* < .001; ICC PHCP = .035		

*Note*: The p‐values in bold type are the values with a significance <.001.

Abbreviations: ADDQoL, Audit of Diabetes‐Dependent Quality of life; BDI‐II, Beck Depression Inventory‐II; CI, confidence interval; DIATEK, Diabetes Knowledge Test; DDS2, Diabetes Distress Scale; HbA1c, glycated haemoglobin; ICC, intraclass correlation; PHCP, primary healthcare practices; STAI‐S, State subscale of the Trait Anxiety Inventory.

## 4 DISCUSSION AND CONCLUSION

### 4.1 Discussion

The INDICA study showed that a multicomponent intervention based on a person‐centred approach for adults with T2DM and their physicians produced improvements in patients' empowerment, knowledge about diabetes and other self‐reported outcomes.^31^ The main objective of this secondary analysis was to identify factors associated with patient empowerment at baseline, before the beginning of the intervention. The results showed that age, diabetes specific‐knowledge and state‐anxiety are significantly associated with patient empowerment.

In previous studies, education has been shown to be significantly associated with empowerment in people with T2DM.^53^, ^54^, ^55^ Higher education levels could facilitate a better understanding of diabetes‐related processes and the awareness of the importance of self‐care in the management of the disease, to prevent or delay severe complications. In our study, however, education level and empowerment were significantly correlated only in the bivariate analysis, but not after controlling for age in the multivariate model. Diabetes‐specific knowledge did show an independent association with empowerment, and these results are consistent with other published studies that found a positive association between diabetes knowledge and patient activation.^56^, ^57^ The basic assumption that actual empowerment is not possible without a certain level of objective knowledge about diabetes, its complications and self‐management strategies is supported by these results. In a classic definition, Funnel et al.^58^ defined patient empowerment as an interactive process of cultivating power through sharing knowledge, expertize and resources. Most people with both type 1 and type 2 diabetes have a need to be listened and want to participate in shared decision‐making,^59^ but for this purpose, both health literacy and diabetes‐specific knowledge are essential. Otherwise, as pointed out by Schulz and Nakamoto,^60^ a sense of self‐efficacy without a corresponding degree of general health literacy and knowledge poses a threat of dangerous health choices. Therefore, PCC interventions should always be built on good‐quality educational programmes adapted to patients' characteristics, a necessary (although not sufficient) component to achieve empowerment.

Regarding the affective dimension of empowerment, only state‐anxiety was found to be a significant (negative) correlate. This result is consistent with several studies across different countries and cultures, showing how high anxiety significantly relates to lower self‐efficacy and patient activation.^61^, ^62^, ^63^ Distress and depression have also shown to be negatively correlated with self‐efficacy.^64^ Although we also observed significant negative associations with patient empowerment in the bivariate analysis, according to the results of the multivariate regression analyses, this association seems to be mediated by state‐anxiety. Negative emotional responses like anxiety and depression could be the result of low levels of self‐efficacy in the management of the disease, producing concerns and uncertainty due to its potential serious complications. Furthermore, these negative emotions may be the result of the inability to integrate psychologically the disease into one's own identity.^23^ On the other hand, diabetes‐unrelated anxiety could act as a barrier to achieve empowerment and good self‐management of the disease. A PCC approach should take into account the patients' affective state, and how it relates to and affects the presence and management of the disease.

Among the nonmodifiable factors, previous studies with T2DM patients also have shown an inverse association between age and empowerment.^65^, ^66^, ^67^, ^68^ Factors that could explain this relationship are the lower levels of health literacy shown by older patients, their preference to be less actively involved in the decision‐making process about their care and more barriers to behavioural changes due to lifestyles established for a long time. Marahrens et al.^69^ assessed factors associated with the preferred role in the decision‐making process of patients with diabetic retinopathy, finding that older patients with low educational attainment were less likely to prefer an active role, and instead, they preferred ophthalmologists to decide. This preference for a more passive role has also been observed among older patients with other health conditions such as prostate cancer,^70^ urine incontinence^71^ or severe mental disorders.^72^

The present study has several limitations. First, it is a secondary analysis of the INDICA study and consequently the choice of the measures was already established before this study. Other potential patient‐, context‐ and psychological‐related variables that can potentially affect patient empowerment (i.e., personality characteristics, social support) have not been assessed. Second, given the observational and cross‐sectional nature of the data, causal inferences cannot be made. The chance to address the direction of the observed association is limited. Third, the sample comes entirely from one Spanish region, and generalization to other contexts requires caution; however, the previously mentioned studies, carried out in very different countries and sociocultural contexts (Europe, Middle and Far East), have shown similar results, reinforcing the generalizability of the findings. Fourth, the use of the short form of the DES, as well as the screening version of the DDS, reduces the scores' variability and may obscure their association with the correlates assessed. Also, the knowledge questionnaire was not previously validated. Fifth, the established inclusion criteria for PHCP, based on feasibility considerations, might be a source of some selection bias in the recruitment process. However, we do not expect practices to be a relevant confounder, since the provision and access to health services are quite homogeneous in a relatively small territory such as the Canary Islands. On the other hand, the low value of the ICC observed indicates that PHCP clustering does not substantially influence the observed associations. Finally, the analysis strategy has been ‘data driven’, and thus results are exploratory and need to be specifically confirmed. This study also has some major strengths, such as its wide sample size and the variety of correlates included in the analyses. Secondary analyses like this are important to make the most of the knowledge obtained in existing studies, even beyond their main aims.

### 4.2 Conclusion

Our study suggests that state‐anxiety, older age and poor diabetes‐specific knowledge are significant correlates of patient empowerment. As empowerment‐based interventions have shown promising results in people with T2DM, our study may be useful to effectively design those interventions and to identify patients who might benefit more. Moreover, further analysis of longitudinal studies is warranted to confirm the direction of the observed association. Likewise, high‐quality longitudinal studies are clearly needed to better understand the causal relationship between patient empowerment and modifiable factors such as anxiety, depression, distress and health‐related quality of life.

### 4.3 Practice implications

Understanding which factors are related to patient empowerment may help clinicians and policymakers to identify high‐risk groups, prioritize resources and target interventions to better support people with T2DM to be actively involved in their own care. Appropriate educational programmes should be at the core of any intervention aimed at increasing empowerment, since objective knowledge is a necessary requisite to achieve this aim. Our results also suggest that anxiety could act as a barrier to empowerment and therefore it should be adequately assessed and managed. Finally, older patients may need special attention, adapting the interventions to their preferences for participation in decision‐making and their health literacy level.

### INDICA TEAM

The INDICA team included the following members: Abraham Pérez de la Rosa (Canary Islands Health Research Institute Foundation, FIISC), Alicia Pareja Ríos (University Hospital of Canary Island), Ana Wägner (Insular University Hospital), Andrés Sifre Perello (Molina Orosa Hospital), Ángela Trinidad Gutiérrez Pérez (Primary Care of Gran Canaria), Antonio Cabrera de León (Ntra Sra de la Candelaria University Hospital), Antonio García Quintana (Dr. Negrín University Hospital), Armando Carrillo Domínguez (Insular University Hospital), Bernardo Eusebio Herrera Domínguez (General de La Palma Hospital), Carlos Sedeño Pérez (Primary Care of Tenerife), Carlos Ramírez Álamo (Primary Care of Gran Canaria), Carmen Daranas Aguilar (Canary Islands Health Research Institute Foundation, FIISC), Carolina Guerra Marrero (Canary Islands Health Research Institute Foundation, FIISC), Cecilia Lobos Soto (Insular University Hospital), Cristina Padrón Pérez (Canary Islands Health Research Institute Foundation, FIISC), Dácil Alvarado Martel (Dr. Negrín University Hospital), Daniel Hernández Obregón (Dr. Negrín University Hospital), Dulce N. Hernández Correa (Primary Care of Gran Canaria), Elsa Espinosa Pozuelo (Diabetes Patient´ association of Tenerife), Elsa Florido Mayor (Canary Islands Health Research Institute Foundation, FIISC), Engracia Pinilla Domínguez (Ntra Sra de la Candelaria University Hospital), Fátima Herrera García (University Hospital of Canary Island), Félix Bonilla Aguiar (Dr. José Molina Hospital), Francisco Cabrera López (Insular University Hospital), Gloria Guerra de la Torre (Primary Care of Gran Canaria), Gregorio Muelas Martín (Dr. Negrín University Hospital), Guillermo Monzón Monzón (Primary Care of Gran Canaria), Héctor de la Rosa Merino (Canary Islands Health Research Institute Foundation, FIISC), Ignacio García Puente (Dr. Negrín University Hospital), Ignacio Llorente Gómez de Segura (Ntra Sra de la Candelaria University Hospital), Isabel García Calcerrada (Ntra Sra de la Candelaria University Hospital), Jacqueline Álvarez Pérez (Canary Islands Health Research Institute Foundation, FIISC), Jorge Federico Aldunate Page (Insular University Hospital), Jose Antonio García Dopico (University Hospital of Canary Island), Juan Andrés Báez Hernández (Primary Care of La Palma), Juan José Pérez Valencia (Primary Care of Tenerife), Julia Charlotte Wiebe (Dr. Negrín University Hospital), Leticia Rodríguez Rodríguez (Canary Islands Health Research Institute Foundation, FIISC), Lidia García Pérez (Canary Islands Health Research Institute Foundation, FIISC), Leopoldo Martín Martín (Hospital General de La Palma), Luis Morcillo Herrera (University Hospital of Canary Island), Marcos Estupiñán Ramírez (Canary Islands Health Service, SCS), María Inmaculada González Pérez (Ntra Sra de la Candelaria University Hospital), María Isabel Visuerte Morales (University Hospital of Canary Island), María Pino Afonso Medina (Dr. Negrín University Hospital), Margarita Roldán Ruano (Primary Care of Gran Canaria), Marta Riaño Ruiz (Insular University Hospital), Marta Tejera Santana (Dr. Negrín University Hospital), Mauro Boronat (Insular University Hospital), Mercedes Lorenzo Medina (Dr. Negrín University Hospital), Miguel Ángel García Bello (Canary Islands Health Research Institute Foundation, FIISC), Miguel Juan Mora García (Primary Care of Gran Canaria), Nayra Pérez Delgado (Ntra Sra de la Candelaria University Hospital), Pablo Pedrianez Martín (Dr. Negrín University Hospital), Pedro de Pablos‐ Velasco (Dr. Negrín University Hospital), Pilar Peláez Alba (La Laguna University), Rafael Valcárcel (Primary Care of Tenerife), Remedios Castro Sánchez (Primary Care of Gran Canaria), Rodrigo Abreu González (Ntra Sra de la Candelaria University Hospital), Rosa Borges Trujillo (Dr. Negrín University Hospital), Sybille Kaiser Giradot (Primary Care of Tenerife) and Víctor Lorenzo Sellarés (University Hospital of Canary Island).

## AUTHOR CONTRIBUTIONS

**Andrea Duarte‐Díaz**: Conceptualization, investigation, methodology, writing – original draft, visualization. **Himar González‐Pacheco**: Formal analysis, writing – review and editing. **Amado Rivero‐Santana**: Conceptualization, methodology, writing – review and editing. **Yolanda Ramallo‐Fariña**: Resources, methodology, writing – review and editing. **Lilisbeth Perestelo‐Pérez**: Conceptualization, methodology, writing – review and editing, supervision. **Wenceslao Peñate**: Conceptualization, writing – review and editing. **Carme Carrion**: Writing – review and editing. **Pedro Serrano‐Aguilar**: Project administration, writing – review and editing.

## CONFLICTS OF INTEREST

The authors declare no conflicts of interest.

## Supporting information

Supporting information.

Supporting information.

## Data Availability

The data that support the findings of this study are available from the corresponding author upon reasonable request.
