# Supplementary material for: Factors associated with patient empowerment in Spanish adults with type 2 diabetes: A cross‐sectional analysis
Source: Health Expect. 2022 Sep 1;25(6):2762–74. doi: 10.1111/hex.13501 (PMC9700189; doi:10.1111/hex.13501)
Supplement: Supplementary file 2 — Supporting information. [file HEX-25--s002.docx]

**Supplemental File 2.** *The Diabetes Knowledge Test (DIATEK)*

To begin with, we expose some issues related to diabetes. Please red each sentence and answer based on what you think is correct.

1. **What do you think Type 2 Diabetes is?**
   1. A disease in which foods containing sugar cannot be eaten.
   2. A disease that occurs as a result of frequent consumption of food rich in sugar.
   3. An inherited disease that is only suffered by those whose parents or grandparents have suffered.
   4. A chronic disease in which blood sugar rises due to failure of insulin secretion and action and can lead to complications if is untreated.
   5. I don’t know.
2. **Which of the following is a risk factor for Type 2 Diabetes?**
   1. To have family members with diabetes
   2. To maintain a sedentary life
   3. Being obese
   4. All of the above
   5. I don't know
3. **Where is insulin produced in our body?**
   1. In the kidney
   2. In the liver
   3. In the pancreas
   4. In the stomach
   5. I don’t know
4. **The test that measures the average level of glucose in the last 3 months is called:**
   1. Basal blood glucose
   2. Glycemic profile
   3. Glycosylated hemoglobin
   4. Self-analysis
   5. I don’t know
5. **Among the general objectives to be achieved by a person with Type 2 Diabetes are:**
   1. No smoking
   2. Maintain blood pressure between 130-139/80-85
   3. Have LDL cholesterol (bad cholesterol) at or below 100mg/dl
   4. All of the above are objective
   5. I don't know
6. **What value of glycosylated hemoglobin indicates a lower risk of developing diabetes-related complications?**
   1. Less than 7%
   2. Between 8% and 9%
   3. More than 10%
   4. Between 11% and 12%
   5. I don’t know
7. **In the Mediterranean diet it is recommended:**
   1. Eat more eggs than fish
   2. Eat red meat almost every day
   3. Eat legumes several times a week
   4. Replace one main meal with only fruit
   5. I don't know
8. **Which of the following cooking methods makes food have more calories and gain more weight?**
   1. Stew
   2. Fried
   3. Boiled
   4. Baked
   5. I don’t know
9. **What type of fat is not recommended to cook in a healthy diet?**
   1. Virgin olive oil
   2. Sunflower oil
   3. light margarine
   4. They are all healthy
   5. I don't know
10. **The main nutrient in rice and potatoes is:**
    1. Protein
    2. Carbohydrate
    3. Fat
    4. Water
    5. I don't know
11. **Of the following foods, which contain the greatest amount of carbohydrates?**
    1. Meat, fish and cheese
    2. Rice, spaghetti and bread
    3. Hazelnuts, oranges and lettuce
    4. Sausages, egg and olive oil
    5. I don't know
12. **Which of the following foods contains the highest proportion of dietary fiber?**
    1. Yogurt
    2. The meat
    3. Vegetables
    4. Eggs
    5. I don't know
13. **Which of these foods can be substituted for 2 pieces of toast?**
    1. One fresh orange juice
    2. Two slices of lean ham
    3. Three Maria cookies
    4. A coffee
    5. I don't know
14. **Which of the following products can be used to sweeten without What affects blood glucose?**
    1. Aspartame
    2. Fructose
    3. Sucrose
    4. Honey
    5. I don't know
15. **The diet for people with Type 2 Diabetes consists of:**
    1. A very special, difficult and expensive diet
    2. It is simply healthy and balanced daily diet
    3. It is very complicated to prepare if you have a family
    4. Consists on not eating carbohydrates (bread, rice, pasta…)
    5. I don't know
16. **It is good to practice physical exercise on a regular basis because:**
    1. Lowers blood glucose and fat levels
    2. Helps lose weight
    3. Improves blood pressure control
    4. All of the above
    5. I don't know
17. **A person with Type 2 Diabetes is recommended to exercise:**
    1. At least once a week
    2. It is better to do nothing so you don’t get injured
    3. At least 30 minutes five days a week
    4. Only when you don't follow the diet or when you eat more than you should
    5. I don't know
18. **The most beneficial for people with Type 2 Diabetes is:**
    1. Combining walking, for example, every day and doing a little weight training at least 2 times a week
    2. Always carrying sugar in your pocket when exercising
    3. Start with very light exercises and finish with stretches, which helps make exercise more beneficial and safer
    4. All of the above
    5. I don't know
19. **The main symptoms of hypoglycemia are:**
    1. Cold sweat and paleness
    2. Tremors and palpitations
    3. Feeling hungry and weak
    4. All of the above
    5. I don't know
20. **Hypoglycemia can be caused by:**
    1. Skip a meal
    2. Perform extraordinary physical exercise
    3. Taking the sugar pill twice by mistake
    4. All of the above
    5. I don't know
21. **To resolve mild-moderate hypoglycemia without loss of consciousness, you should take:**
    1. A glass of water with 2 tablespoons of sugar, wait 10-15 minutes and measure glucose again, then rest
    2. A soft drink sugar free or Light
    3. Quickly eat as much food as you can
    4. Inject Glucagon
    5. I don't know
22. **Which of the following health problems appear as a result of poorly controlled diabetes?**
    1. Blindness
    2. Dialysis
    3. Amputation
    4. All of the above
    5. I don't know
23. **The presence of kidney damage due to diabetes:**
    1. No major consequences
    2. It is impossible to prevent it
    3. It is very important to prevent and treat it to avoid getting into dialysis or a kidney transplant
    4. All people with Type 2 Diabetes have it
    5. I don't know
24. **To prevent the appearance of chronic complications associated with diabetes (heart attack, blindness, dialysis, amputation) it is necessary:**
    1. Adequate control of diabetes
    2. Adequate control of blood pressure and LDL cholesterol (bad cholesterol)
    3. No smoking
    4. All of the above
    5. I don't know
25. **The appearance of complications in the feet can be seen increased by the following factors:**
    1. Smoking
    2. Have reduced mobility
    3. Have a loss of sensation in the feet
    4. All of the above
    5. I don't know
26. **To avoid foot complications, you should:**
    1. Have good control of diabetes, blood pressure, and heart rate.
    2. Cholesterol
    3. Inspect feet daily, including the bottom of the foot and between the fingers
    4. No smoking
    5. All are true
    6. I don't know
27. **The foot of the person with Type 2 Diabetes has a higher risk of:**
    1. Dry skin
    2. Decreased sensitivity (to temperature, to touch…)
    3. Decreased blood circulation
    4. All of the above
    5. I don't know
28. **Select to the correct answer:**
    1. Insulin exists in pill form
    2. Insulin can be frozen
    3. Insulin can be injected into the abdomen, arms, thighs, or buttocks
    4. Cold insulin has more effect
    5. I don't know
29. **Mark the correct answer:**
    1. Insulin is always given after a meal to lower blood glucose after eating
    2. The pills only serve to delay the administration of the insulin, because the person with Type 2 Diabetes will need it anyway
    3. Both pills and insulin can be used alone or in combination to help control diabetes
    4. Insulin should not be used for Type 2 Diabetes; insulin is needed only for children with this type of diabetes.
    5. I don't know
30. **Which of the following is a correct statement?**
    1. It is better for friends and family not to know that a person has Type 2 Diabetes
    2. With Type 2 Diabetes you can go out to eat
    3. With Type 2 Diabetes you cannot travel
    4. With Type 2 Diabetes you can never eat sweets
    5. I don't know
